# Associations of Serum Total 25OHD, 25OHD3, and epi-25OHD3 with Insulin Resistance: Cross-Sectional Analysis of the National Health and Nutrition Examination Survey, 2011–2016

**DOI:** 10.3390/nu14173526

**Published:** 2022-08-26

**Authors:** Meiling Zhou, Ruixue Huang

**Affiliations:** Department of Occupational and Environmental Health, Xiangya School of Public Health, Central South University, Changsha 410078, China

**Keywords:** exposure, insulin resistance, vitamin D

## Abstract

Background: Vitamin D may have a role in insulin sensitivity. However, the data on the association between various metabolites of Vitamin D and insulin-related parameters have been limited. Methods: We identified 6026 adults aged 20–80 years who participated in the 2011–2016 National Health and Nutrition Examination Survey (NHANES). Serum total 25OHD, 25OHD3, and epi-25OHD3, fasting glucose, insulin, and HOMA2-IR were obtained from the NHANES data. The association between serum Vitamin D-related values and insulin resistance was analyzed using a generalized linear model. For risk analysis, multifactorial logistic regression was used. Results: The median total 25-hydroxyvitamin D level, 25-hydroxyvitamin D3 level, and 3-epi-25-hydroxyvitamin D3 level were 62.5 nmol/L, 58.8 nmol/L, and 3.3 nmol/L, respectively. After adjustment for sex, age, race, ethnicity, and education status, the ORs for the insulin resistance of participants of total 25-hydroxyvitamin D, 25-hydroxyvitamin D3, and 3-epi-25-hydroxyvitamin D3 were 0.32 (95% CI 0.24, 0.43), 0.34 (95% CI 0.26, 0.44), and 0.64 (95% CI 0.53, 0.77), respectively. After an adjustment for body mass index, diabetes, and drinking and smoking, the ORs for the insulin resistance of the participants for total 25-hydroxyvitamin D, 25-hydroxyvitamin D3, and 3-epi-25-hydroxyvitamin D3 were 0.56 (95% CI 0.40, 0.78), 0.63 (95% CI 0.46, 0.85), and 0.99 (95% CI 0.80, 1.24), respectively. Conclusions: Our study provides suggestive evidence for the association between Vitamin D concentrations and a lower risk of insulin resistance. Evidence from larger and more adequately powered cohort studies is needed to confirm our results.

## 1. Introduction

Vitamin D is one of the vitamins necessary for the human body to maintain life and health [1]. It is a fat-soluble vitamin that plays an important role in maintaining calcium homeostasis in the body [2]. Vitamin D deficiency has been reported worldwide [3]; when plasma 25-(OH)D3 concentration is <30 ng/mL, it can be judged as Vitamin D deficiency. In addition, many studies have shown that Vitamin D plays an important role in maintaining the metabolic balance of calcium and bone formation in the body [4]. However, in recent years, more and more attention has been paid to its potential extraosseous effects [5]. Many studies have found that Vitamin D has immunomodulatory effects; besides this, vitamin D deficiency may be associated with low-grade inflammatory states, obesity, metabolic syndrome, and essential hypertension [6].

Recent studies have shown that in addition to protecting bones, vitamin D is also closely related to the occurrence of diabetes [7,8]. Adequate vitamin D has a certain effect on promoting insulin secretion and reducing insulin resistance [9]. Research has shown that diabetes has become one of the major chronic non-communicable diseases affecting the health of global residents [10]. The latest data from the International Diabetes Federation (IDF) show that the total number of people with diabetes worldwide will reach 537 million in 2021. It is estimated that by 2045, the number of people with diabetes worldwide could be as high as 783 million [11,12]. Insulin resistance is a mechanism in which insulin-dependent cells (such as adipocytes) respond inappropriately to insulin. The etiological pathway is not yet clear, and the etiology involves the combined action of environmental and genetic factors [13]. In addition, insulin resistance is a common underlying physiological abnormality associated with type 2 diabetes [14]. Without the effective control of insulin resistance, it is suggested that diabetes will develop eventually.

Previous cross-sectional clinical studies have indicated that even a low serum vitamin D level is associated with a decreased insulin resistance risk in adults and children [15,16]. In terms of mechanism, vitamin D may have a favorable effect on insulin sensitivity through a series of mechanisms, including an increase in the transcriptional activation and expression of insulin receptor genes, promoting basal and insulin-stimulated glucose oxidation, and thereby improving insulin sensitivity [17,18]. Vitamin D3 supplementation has been proven to improve insulin sensitivity in subjects with impaired fasting glucose [19]. Moreover, observational studies on animal models have shown that Vitamin D levels can promote the synthesis and secretion of insulin in the pancreas of mice [20]. In conclusion, there is a close relationship between insulin resistance and vitamin D. The potential effects of both were found to provide measures to reduce the incidence of diabetes. However, data on the association between insulin-related parameters and various metabolites of Vitamin D—in particular, the total 25-hydroxyvitamin D (25OHD) level, 25-hydroxyvitamin D3(25OHD3) level, and 3-epi-25-hydroxyvitamin D3 (epi-25OHD3)—have been limited. This study aimed to explore the relationship between insulin resistance and vitamin D metabolites through multivariate logistic regression analysis and risk analysis using a NHANES database that is available online.

## 2. Materials and Methods

### 2.1. Study Population

The data used in this study were extracted from NHANES [21]. We recruited adults (>18 years of age) from the 2011–2016 period of NHANES. The total sample size was 29,903, including 6026 samples with detection data for insulin resistance and Vitamin D levels. The specific exclusion steps were conducted as follows: the remaining sample size after removing the insulin missing values from the total sample size was 8890; next, 2864 missing values for vitamin D and other related variables were removed; finally, a remaining sample size of 6026 was included in our study for follow-up research. As required by the Institutional Review Board of the National Center for Health Statistics, the NHANES participants provided written informed consent [22].

### 2.2. The Determination of Vitamin D Metabolites

According to the NHANES data collection guidelines, serum specimens were collected using regular red-top or serum-separator Vacutainers™. Ultra-high-performance liquid chromatography–tandem mass spectrometry (UHPLC-MS/MS) was used to quantitatively detect 25-hydroxyvitamin D3 (25OHD3), 3-epi-25-hydroxyvitamin D3 (epi-25OHD3), and total 25-Hydroxyvitamin D (total 25OHD) in human serum. The analytes were chromatographically separated generally on one of three pentafluorophenyl (PFP) columns (Thermo Scientific, Waltham, MA, USA, Hypersil GOLD PFP 2.1 × 100 mm, 1.9 µm particle size column, Phenomenex, Torrance, CA, USA, Kinetex PFP 2.1 × 100 mm, 1.7 µm, or Sigma-Aldrich, St. Louis, MO, USA, Ascentis Express F5, 2.1 × 150 mm, 2.7 μm). Detection was performed using a triple quadrupole tandem mass spectrometer (Thermo TSQ Vantage system) using atmospheric pressure chemical ionization in the positive ion mode. Quantitation was accomplished by comparing the response ratio in the unknown, with a response ratio of a known amount of analyte in a calibrator solution. Response ratios are based on the peak area of the analyte divided by the peak area of the internal standard.

Because 25OHD is very stable, serum samples can be frozen at −20 °C to −70 °C for years before analysis. The limits of detection (nmol/L) were calculated as follows: 4.88 and 1.88 for 25OHD3 and epi-25OHD3.

### 2.3. Determination of Fasting Glucose, Insulin, and HOMA-IR

Before performing insulin-related experiments such as insulin and fasting glucose, participants were required to fast overnight. Serum was collected in Red Top Tubes. Collected samples can be stored at −20 °C for two months. Serum insulin was measured using an immunoenzymometric assay, with the TOSOH AIA-900 Chemistry Analyzer in University of Missouri-Columbia.

In addition, blood was collected from fasting participants via venipuncture. Fasting glucose was measured using a hexokinase-mediated reaction and a Roche/Hitachi Cobas C 501 Chemistry Analyzer in University of Missouri-Columbia. Ultimately, fasting glucose and insulin values were used to calculate the HOMA-IR value as follows [23]:

HOMA-IR = fasting blood glucose (mmol/L) × serum insulin (μU/mL)/22.5

Many studies have different cut-off values for HOMA-IR. There were reports that the HOMA-IR values were divided into quarters for analysis [24]. In addition, some studies showed that the HOMA-IR values were set based on the difference of the BMI values [25]. We mainly refer to clinical criteria for grouping. The cut-off value was set as follows [26]:

Insulin resistance values (HOMA-IR) of ≥2.00 were considered to indicate insulin resistance [27].

### 2.4. Other Covariates

Questionnaire information included general demographic characteristics and general life behaviors. General demographic characteristics included sex, age, race, and education status. General behavioral characteristics included drinking, smoking, and physical activities. The information was based on participants’ self-reports. In addition, body mass index (BMI) was calculated by dividing the weight (kg) by height (meters) squared. Cholesterol was measured using the Beckman UniCel^®^ DxC 800 Synchron and Beckman UniCel^®^ DxC 660i Synchron Access Clinical Systems. Creatinine (urine) and albumin (urine) were measured using an Enzymatic Roche Cobas 6000 Analyzer and a fluorescein immunoassay via a SequoiaTurner Digital Fluorometer, Model 450, respectively. As for dietary intake, the nutritional assessment component of the current NHANES includes a 24 h dietary recall interview for participants of all ages. Dietary recall interviews were conducted in person by trained dietary interviewers fluent in Spanish and English. The setting of the interview was a private room in the Mobile Examination Center (MEC). Each MEC dietary interview room contained a standard set of measuring guides. According to the diagnostic criteria for diabetes given by the American Diabetes Association, the reference level for fasting blood glucose is: FPG (fasting plasma glucose) of ≥126 mg/dL (7.0 mmol/L). Fasting is defined as having no caloric intake for at least 8 h. Therefore, in this survey, type 2 diabetes was defined as a fasting blood glucose level of ≥7.0 mmol/L or a self-reported diabetes diagnosis.

### 2.5. Statistical Analysis

In this study, the analysis software used to process the data was SPSS (v. 25.0; IBM Corp., Armonk, NY, USA) and R software (v. 4.0.3; R Foundation for Statistical Computing, Vienna, Austria). The data analysis standard was a two-sided test, and the test level was α = 0.05. Because the distribution of total 25-hydroxyvitamin D, 25-hydroxyvitamin D3, and 3-epi-25-hydroxyvitamin D3 concentrations were right skewed, we log-transformed the data. Since the data did not conform to a normal distribution, these samples were described using the median and interquartile range. The relationships between the participant characteristics and the 25-hydroxyvitamin D concentration (total 25-hydroxyvitamin D, 25-hydroxyvitamin D3, and 3-epi-25-hydroxyvitamin D3) were evaluated using the Wilcoxon rank-sum test or the Kruskal–Wallis test. The relationship between the 25-hydroxyvitamin D concentration and insulin resistance was not linear. Therefore, the data were analyzed using a generalized linear model. As for risk analysis, we used a multifactorial logistic regression. The odds ratios (ORs) of HOMA-IR and 25-hydroxyvitamin D concentration were calculated at the 20th and 80th percentiles. Furthermore, we also calculated the odds ratio of the HOMA-IR and 25-hydroxyvitamin D concentrations at the 30th with 70th percentiles. The 25-hydroxyvitamin D concentration was divided into tertiles in order to evaluate non-linear relationships, the lowest of which was compared with the other two.

The logistic regression analysis method was used. Furthermore, the logistic regression model for 25-hydroxyvitamin D concentration and HOMA-IR was adjusted. We employed four tuning models—the first model was for sex, age, race and ethnicity, and education status; the second model further adjusted for body mass index, diabetes, and drinking and smoking, while the third model further adjusted for creatinine (urine), albumin (urine), and cholesterol; the last model further adjusted for carbohydrate, protein, fat, dietary fiber, vitamin D, and calcium. Moreover, we constructed four models to perform a risk analysis. Consistent with the logistic regression model described earlier, the same four model adjustments were made.

We estimated the proportions of the geometric mean vitamin D concentrations by comparing the subgroups defined according to sex, age, race and ethnicity, education, body mass index, diabetes, smoking status, and alcohol use between participants with and without insulin resistance. The purpose was to assess the consistency of the findings according to the characteristics of the participants.

## 3. Results

A total of 6026 participants were included in the study; 54.0% were male and 46.0% were female. The participants age range was 20–80 years. Non-Hispanics comprised the largest number of participants, at 62.1% of the total. Among these participants, a BMI greater than 30 accounted for the majority, at 37.8%. It is worth noting that 13.1% of people self-reported or were diagnosed with diabetes. The number of participants who developed insulin resistance (HOMA-IR value ≥ 2.0) was more than half of the total, accounting for 60.8%.

In the analyzed 2011–2016 NHANES data, the median total 25-hydroxyvitamin D level, 25-hydroxyvitamin D3 level, and 3-epi-25-hydroxyvitamin D3 level were 62.5 nmol/L, 58.8 nmol/L, and 3.3 nmol/L (Table 1), respectively. Among the population characteristics analyzed, participants who were male, older than 60, non-Hispanic, BMI < 25, educated at least at high school level, smokers, diabetics, and those who did not develop insulin resistance had higher levels of total 25-hydroxyvitamin D, 25-hydroxyvitamin D3, and 3-epi-25-hydroxyvitamin D3. In addition, the participants with albumin (urine) <5.6, creatinine (urine) <0.81, cholesterol >5.25, had higher concentrations of total 25-hydroxyvitamin D, 25-hydroxyvitamin D3, and 3-epi-25-hydroxyvitamin D3 (Table 1).

The ORs for the insulin resistance of participants of total 25-hydroxyvitamin D, 25-hydroxyvitamin D3, and 3-epi-25-hydroxyvitamin D3, respectively, were 0.32 (95% CI 0.24, 0.43), 0.34 (95% CI 0.26, 0.44), and 0.64 (95% CI 0.53, 0.77) separately, after adjustment for sex, age, race and ethnicity, and education status (Table 2; model 1); and 0.56 (95% CI 0.40, 0.78), 0.63 (95% CI 0.46, 0.85), and 0.99 (95% CI 0.80, 1.24) after adjustment for body mass index, diabetes, and drinking and smoking (Table 2; model 2). In addition, they were 0.57 (95% CI 0.41, 0.80), 0.64 (95% CI 0.47, 0.86), and 1.00 (95% CI 0.82, 1.26) after adjustment for creatinine (urine), albumin (urine), and cholesterol (Table 2; model 3); they were 0.61 (95% CI 0.43, 0.86), 0.67 (95% CI 0.49, 0.91), and 1.05 (95% CI 0.92, 1.18) after further adjustment for carbohydrate, protein, fat, dietary fiber, vitamin D, and calcium (Table 2; model 4).

The ORs for the insulin resistance of participants in the 70th versus 30th percentiles of total 25-hydroxyvitamin D, 25-hydroxyvitamin D3, and 3-epi-25-hydroxyvitamin D3 were, respectively, 0.34 (95% CI 0.25, 0.46), 0.35 (95% CI 0.27, 0.46), and 0.51 (95% CI 0.41, 0.63) separately, after adjustment for sex, age, race and ethnicity, and education status (Table 2; model 1), and 0.58 (95% CI 0.41, 0.82), 0.65 (95% CI 0.47, 0.90), and 0.81 (95% CI 0.63, 1.04) after adjustment for body mass index, diabetes, and drinking and smoking (Table 2; model 2). In addition, they were 0.58 (95% CI 0.40, 0.83), 0.66 (95% CI 0.48, 0.92), and 0.82 (95% CI 0.64, 1.05) after adjustment for creatinine (urine), albumin (urine), and cholesterol (Table 2; model 3), and 0.62 (95% CI 0.43, 0.90), 0.71 (95% CI 0.51, 0.99), and 0.85 (95% CI 0.66, 1.10) after adjustment for carbohydrate, protein, fat, dietary fiber, vitamin D, calcium (Table 2; model 4). The 80th versus 20th percentiles of total 25-hydroxyvitamin D,25-hydroxyvitamin D3, and 3-epi-25-hydroxy Vitamin D3 were also the same as above (Table 2).

To further demonstrate the relationship between insulin resistance and vitamin D, we performed a tertile analysis of the total 25-hydroxyvitamin D, 25-hydroxyvitamin D3, and 3-epi-25-hydroxyvitamin D3. The ORs for insulin resistance decreased in the four adjusted models, decreasing with increasing Vitamin D concentrations, and the differences were statistically significant (Table 3).

**Figure 1:**
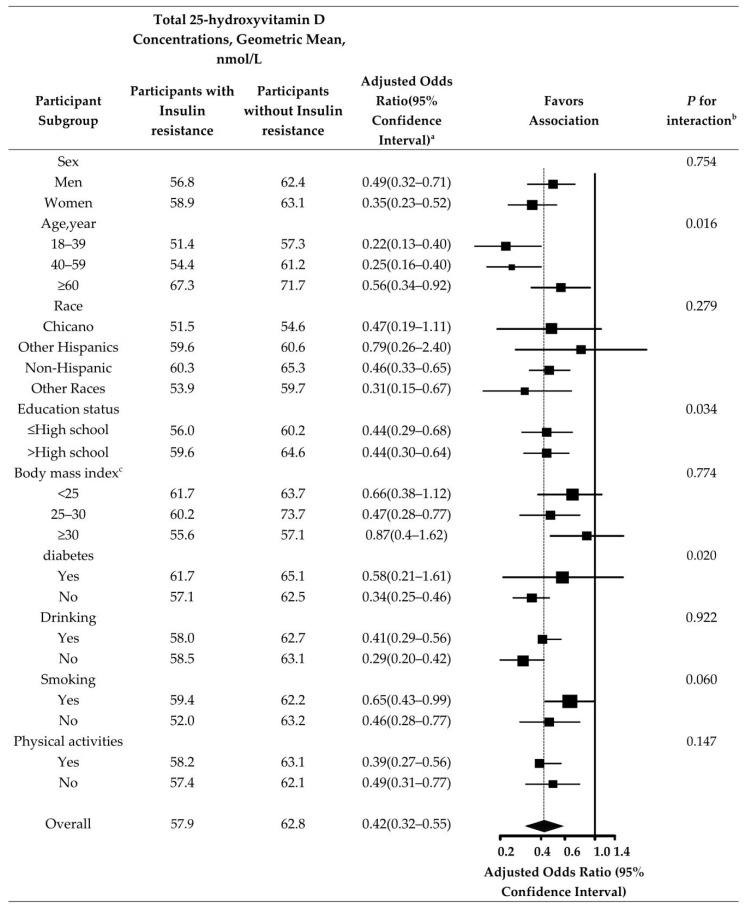
Total 25-hydroxyvitamin D Concentrations Comparing Participants With insulin resistance. ^a^ Odds Ratio (95% confidence interval) is also adjusted for creatinine (urine), albumin (urine), and cholesterol. ^b^ *P* for interaction based on log-transformed total 25-hydroxyvitamin D concentrations. ^c^ Body mass index is calculated as weight in kilograms divided by height in meters squared.

The negative association between total 25-hydroxyvitamin D and insulin resistance after adjustment for creatinine (urine), albumin (urine), and cholesterol was consistent in most subgroups, and was somewhat stronger in female, younger, higher education, and never-smoking and never-drinking participants (Figure 1).

**Figure 2:**
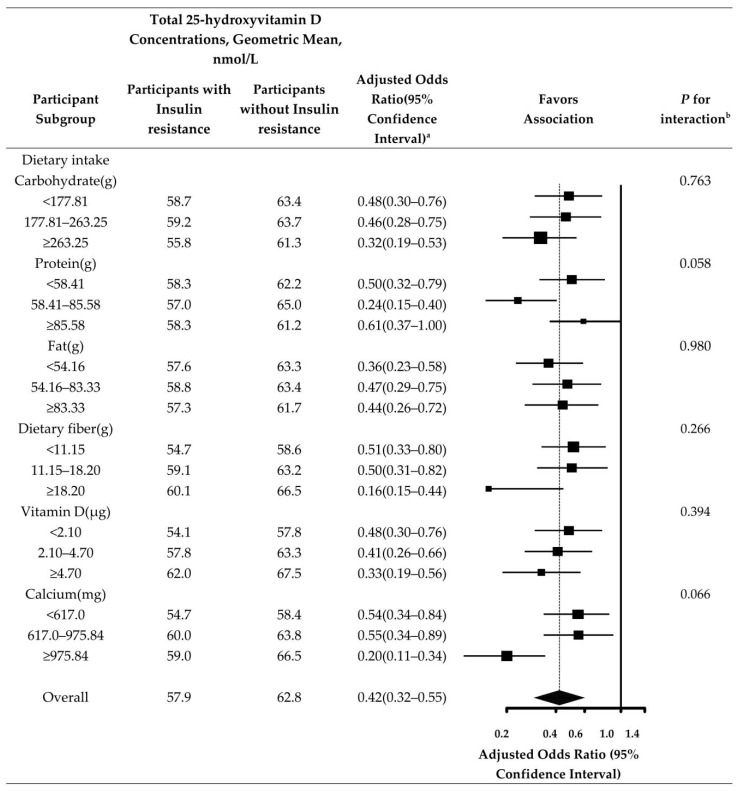
Total 25-hydroxyvitamin D Concentrations Comparing Participants With insulin resistance. ^a^ Odds Ratio (95% confidence interval) is also adjusted for creatinine (urine), albumin (urine), and cholesterol. ^b^ *P* for interaction based on log-transformed total 25-hydroxyvitamin D concentrations.

In addition, we analyzed the relevant nutrients (protein, carbohydrate, fat, dietary fiber, vitamin D, and calcium) in the participants’ diets. The negative correlation between total 25 hydroxyvitamin D and insulin resistance after adjustment for creatinine (urine), albumin (urine), and cholesterol was consistent in most subgroups. Additionally, it was more obvious in those who consumed more than 236.25 g of carbohydrates, less than 54.16 g of fat, more than 18.2 g of dietary fiber, and more than 4.7 μg of vitamin D per day (Figure 2).

## 4. Discussion

In this study, the NHANES database was used to expose the relationship between insulin resistance and vitamin D levels. The most important result obtained from the analysis was a significant negative correlation between vitamin D levels and insulin resistance index, which was consistent with the results reported by Danting Li et al. [28], Yun Gao et al. [29], and Mahtab, Niroomand et al. [30]. However, several studies have found contradictory conclusions, such as the results of Pilz S. et al. [31] and Heshmat et al. [32]. There are many reasons for the inconsistent results. For example, due to differences in research samples, the analysis results are inconsistent, including differences in sample size, population, and region. In addition, the cut-off value of the insulin resistance index in this data analysis is mainly 2.00 [27]. Regarding the cut-off value of HOMA-IR, many reference range divisions have been reported. For example, Chieh-An Lin et al. set different cut-off values according to different ages [33]. Some studies have divided the cutoff value of HOMA-IR according to the disease status, such as whether there is fatty liver [34]. In addition, C H Lee et al. used a prospective study to determine the optimal cutoff for HOMA-IR [35]. In our analysis, we mainly used the cut-off values commonly used in clinical diagnostic criteria for grouping [26]. The included participants were divided into two groups with or without insulin resistance for analysis.

Subgroup analyses showed that the relationship between Vitamin D and insulin resistance varied across age (*p* = 0.016), education status (*p* = 0.034), and diabetes (*p* = 0.020). The results showed that Vitamin D is a protective factor for insulin resistance at different ages. The younger the age, the stronger the protective effect of Vitamin D on insulin resistance; its protective effect will weaken with increasing age. This is consistent with the findings of G Paolisso et al. [36]. The possible reasons are as follows: among young people, the body’s immune system and metabolic mechanisms are active, and insulin sensitivity is higher than that of the aging population [37]. In contrast, for older people, aging increases the risk of diabetes, whereas diabetes is linked with insulin resistance [38,39]. Our data demonstrated that Vitamin D was a protective factor for insulin resistance in different education status. The higher the cultural level, the stronger the protective effect of Vitamin D on insulin resistance. This is consistent with the findings of Marilyn Tseng et al. [40]. The higher the educational level, the more comprehensive the knowledge received, the greater the concern about one’s own health. Greater awareness of physical examination and medical treatment led to timely detection of physical conditions, and a reduction in the incidence of some common diseases such as diabetes. In addition, insulin resistance is a major factor in the development of diabetes. Most people with type 2 diabetes have insulin resistance [41]. This is consistent with the results of our analysis. The protective effect of vitamin D on insulin resistance was stronger in participants without diabetes. In Figure 2, we can also see that dietary nutrient intake also has an effect on the relationship between vitamin D and insulin resistance, although the interaction between the two is not significant. Increased intake of carbohydrates, dietary fiber and calcium, and decreased intake of fat may reduce insulin resistance; this is consistent with the results of Emilia Papakonstantinou et al. [42] and Hana Kahleova o et al. [43].

The effect of vitamin D on insulin sensitivity has been demonstrated in vitro, in vivo (cellular and animal), and in the human population [20,44,45,46]. Our results have demonstrated that participants who have higher Vitamin D levels are associated with a lower risk of insulin resistance. Our results support the findings of previous studies [47,48,49]. Phyllis A Nsiah-Kumi et al. suggested that a high oral dose of Vitamin D3 improves insulin sensitivity in subjects with impaired fasting glucose [19]. Nsiah-Kumi et al. surveyed 198 American adolescents and found that Vitamin D was inversely associated with HOMA-IR [50]. Collectively, these results suggest that increasing the supplement of Vitamin D could reduce the occurrence of insulin resistance, thereby reducing the incidence of diabetes.

In recent years, many studies have reported on the mechanism between insulin resistance and Vitamin D. Sha Tao et al. showed that Vitamin D deficiency causes insulin resistance by causing oxidative stress in hepatocytes [51]. In addition, there is evidence that Vitamin D receptors are expressed by pancreatic β cells; therefore, vitamin D is required for normal insulin secretion [51,52]. Beyond that, Vitamin D can stimulate insulin receptor expression on target tissues through its interaction with skeletal muscle VDRs (Vitamin D receptors), resulting in increased insulin sensitivity [51]. There is also evidence that Vitamin D may be associated with obesity and muscle mass. Vitamin D reduces obesity, thereby indirectly increasing insulin sensitivity by improving muscle mass [44,53]. Finally, Vitamin D and calcium can play a role in the secretion of insulin by pancreatic cells. Vitamin D deficiency leads to an increase in PTH (parathyroid hormone) secretion, resulting in a continuous increase in intracellular calcium levels, which can lead to insulin resistance [54,55,56]. 

Moreover, our research also showed that female, younger, more educated, BMI < 25, non-smoking, and non-drinking participants had more Vitamin D levels and were less likely to develop insulin resistance. The results of a double-blind randomized clinical trial by Mahtab Niroomand et al. suggest that high-dose Vitamin D improves insulin sensitivity [30]. A review by Anastassios G Pittas. et al. showed that the results of many trials are consistent with substantial evidence from observational studies for a protective role of Vitamin D in modulating diabetes risk [7].

In summary, our study analyzed the association between Vitamin D levels and insulin resistance risk from the perspective of Vitamin D’s three critical metabolites, serum 25OHD2, 25OHD3, and epi-25OHD3. Obviously, there are significant negative correlations between insulin resistance and serum 25OHD2, 25OHD3, and epi-25OHD3. Our study also found vitamin insufficiency—in particular, 25ODH3 insufficiency—was highly common in some populations, including Chicanos, and this needs further preventative attention. Although the correlation was reduced after adjusting for confounding factors, both are statistically significant. However, this study also has certain shortcomings, such as the influence of dietary intake not being considered when the variables were included in the analysis. In addition, this study is a cross-sectional study; only correlations can be obtained, and there is no obvious causal relationship. Because the research method used in this study is a cross-sectional study, if we want to understand the relationship between insulin resistance and vitamin D, we may need to use cohort studies or conduct animal experiments. Another reason is that this study is based on NHANES population data over 2011–2016, which is not supported by the updated data.

## 5. Conclusions

In conclusion, this population-based cross-sectional study demonstrates that vitamin D (total 25-hydroxyvitamin D, 25-hydroxyvitamin D3, and 3-epi-25-hydroxyvitamin D3) is inversely associated with insulin resistance. This suggests that interventions on improving 25ODH, 25ODH3, and epi-25ODH levels may improve insulin resistance. However, clinical trials such as a RCT (random control trial) and a cohort study with a large population are needed for further evaluation and to confirm the association.

## Figures and Tables

**Table 1:** Concentrations of 25-hydroxyvitamin D according to Participant Characteristics.

		25-Hydroxyvitamin D Concentration, Median (IQR), nmol/L			*p* Value ^a^		
Characteristics	No. (%) ^a^	Total 25-Hydroxyvitamin D	25-Hydroxyvitamin D3	3-epi-25-Hydroxyvitamin D3	Total 25-Hydroxyvitamin D	25-Hydroxyvitamin D3	3-epi-25-Hydroxyvitamin D3
Overall	6026	62.5 (45.7–81.1)	58.8 (41.6–77.2)	3.3 (2.0–5.0)			
Sex					0.027	0.021	<0.001
Men	3252 (54.0)	63.1 (47.2–80.9)	59.4 (43.1–77.3)	3.4 (2.1–5.2)			
Women	2875 (46.0)	61.6 (43.8–81.4)	57.9 (40.2–77.1)	3.2 (1.9–4.8)			
Age, year					<0.001	<0.001	<0.001
18–39	1663 (27.6)	56.9 (42.0–71.2)	54.5 (39.4–69.1)	3.0 (1.9–4.3)			
40–59	2348 (39.0)	60.2 (43.8–77.5)	56.4 (40.0–73.5)	3.1 (1.9–4.7)			
≥60	2015 (33.4)	73.3 (53.4–92.4)	67.2 (46.3–87.4)	3.9 (2.4–6.0)			
Race					<0.001	<0.001	<0.001
Chicano	834 (13.8)	53.7 (41.0–68.4)	50.4 (37.9–64.9)	2.9 (1.8–4.1)			
Other Hispanics	653 (10.8)	61.1 (49.3–73.9)	57.4 (45.5–70.5)	3.3 (2.2–4.4)			
Non-Hispanic	3740 (62.1)	66.2 (47.1–85.4)	62.2 (42.4–81.5)	3.5 (2.1–5.4)			
Other Races	799 (13.3)	58.3 (43.6–77.7)	54.0 (39.7–73.3)	3.0 (1.9–4.4)			
Body mass index					<0.001	<0.001	<0.001
<25	1791 (29.7)	65.8 (48.0–85.2)	61.9 (44.0–82.2)	3.4 (2.2–5.3)			
25≤30	1956 (32.5)	64.0 (48.0–82.0)	60.3 (44.7–78.6)	3.4 (2.2–5.3)			
≥30	2279 (37.8)	68.8 (42.5–76.6)	54.4 (37.4–71.7)	3.0 (1.8–4.6)			
Drinking					0.531	0.052	<0.001
Yes	4290 (71.2)	62.8 (45.8–80.9)	59.5 (42.2–77.4)	3.4 (2.1–5.1)			
No	1736 (28.8)	61.7 (45.6–82.1)	56.8 (40.1–76.8)	3.1 (1.9–4.7)			
Diabetes					0.009	0.146	0.856
Yes	787 (13.1)	64.9 (46.7–84.8)	58.3 (39.9–77.0)	3.3 (2.0–4.9)			
No	5239 (86.9)	62.1 (45.5–80.8)	58.9 (41.9–77.3)	3.3 (2.0–5.0)			
Insulin resistance					<0.001	<0.001	<0.001
Yes	3664 (60.8)	60.5 (44.4–78.5)	56.1 (40.1–74.7)	3.2 (2.0–4.9)			
No	2362 (39.2)	65.8 (48.6–84.9)	62.3 (44.6–81.3)	3.4 (2.2–5.2)			
Smoking					0.069	0.204	<0.001
Yes	2587 (42.9)	63.9 (46.2–82.8)	60.0 (41.8–78.9)	3.5 (2.1–5.3)			
No	3439 (57.1)	61.4 (45.4–79.8)	57.7 (41.5–76.3)	3.1 (2.0–4.8)			
Creatinine (urine) mg/dL					<0.001	<0.001	<0.001
<0.81	1971 (32.7)	64.8 (49.3–82.9)	61.1 (45.8–79.7)	3.4 (2.2–5.2)			
0.81–1.44	2038 (33.8)	62.1 (45.8–80.1)	58.6 (41.7–76.4)	3.3 (2.0–5.0)			
≥1.44	2017 (33.5)	60.8 (42.7–80.4)	56.0 (37.8–75.5)	3.2 (1.8–4.8)			
Albumin (urine) (mg/L)					<0.001	<0.001	<0.001
<5.6	1999 (33.2)	67.0 (50.3–86.8)	62.7 (46.0–82.3)	3.5 (2.2–5.3)			
5.6–13.2	2014 (33.4)	62.6 (45.5–81.2)	58.9 (41.7–77.8)	3.3 (2.0–4.9)			
≥13.2	2013 (33.4)	58.1 (42.1–74.6)	54.6 (38.2–71.5)	3.1 (1.9–4.7)			
Cholesterol (mmol/L)					<0.001	<0.001	0.06
<4.4	1983 (32.9)	60.2 (44.2–77.5)	56.8 (40.7–73.7)	3.18 (2.0–4.9)			
4.4–5.25	2000 (33.2)	63.5 (45.9–82.4)	60.0 (41.9–78.4)	3.4 (2.1–5.1)			
≥5.25	2043 (33.9)	63.9 (63.9–83.5)	59.8 (42.4–79.3)	3.3 (2.1–4.9)			
Education status					<0.001	<0.001	<0.001
<High school	1335 (22.2)	58.5 (43.9–75.3)	54.6 (40.2–71.0)	3.0 (1.9–4.5)			
High school	1300 (21.6)	60.8 (43.9–80.9)	57.3 (39.7–76.6)	3.2 (2.0–4.9)			
>High school	3391 (56.3)	64.7 (47.5–83.0)	60.9 (43.079.4)	3.4 (2.1–5.3)			
Physical activities					0.093	<0.001	<0.001
Yes	3896 (64.7)	62.8 (46.7–80.9)	59.7 (43.2–77.8)	3.4 (2.1–5.2)			
No	2130 (35.3)	61.9 (44.2–82.0)	56.8 (38.5–76.1)	3.1 (1.8–4.7)			
Dietary intake							
Carbohydrate (g)					0.001	0.117	0.359
<177.81	2006 (33.3)	63.0 (45.1–82.8)	58.1 (40.5–78.4)	3.3 (2.0–5.0)			
177.81–263.25	2013 (33.4)	64.2 (46.4–82.8)	60.0 (42.3–78.6)	3.3 (2.0–5.2)			
≥263.25	2007 (33.3)	61.0 (45.5–78.2)	58.3 (42.5–75.3)	3.3 (2.1–4.8)			
Protein (g)					0.753	0.112	0.096
<58.41	2006 (33.3)	62.5 (44.5–82.4)	57.9 (40.2–77.4)	3.2 (1.9–4.9)			
58.41–85.58	1981 (32.9)	62.5 (46.2–81.5)	59.2 (42.4–78.4)	3.3 (2.1–5.1)			
≥85.58	2039 (33.8)	62.4 (46.3–79.7)	59.1 (42.8–76.0)	3.3 (2.1–4.9)			
Fat (g)					0.149	0.178	0.287
<54.16	2006 (33.3)	62.4 (44.8–81.4)	57.4 (40.3–76.7)	3.1 (2.0–4.9)			
54.16–83.33	2013 (33.4)	62.9 (46.4–82.9)	59.3 (42.2–78.8)	3.3 (2.1–5.0)			
≥83.33	2007 (33.3)	62.1 (45.8–79.6)	59.4 (42.7–76.1)	3.3 (2.1–5.1)			
Dietary fiber (g)					<0.001	<0.001	<0.001
<11.15	2004 (33.3)	58.7 (42.1–78.0)	54.4 (36.9–72.8)	3.1 (1.8–4.7)			
11.15–18.20	2005 (33.3)	63.3 (46.4–82.2)	59.5 (42.6–78.7)	3.3 (2.1–5.0)			
≥18.20	2017 (33.4)	64.8 (48.8–83.0)	61.2 (45.5–79.2)	3.5 (2.2–5.2)			
Vitamin D (μg)					<0.001	<0.001	<0.001
<2.10	1976 (32.8)	57.8 (41.8–77.7)	53.4 (37.3–72.8)	3.1 (1.8–4.7)			
2.10–4.70	2030 (33.7)	63.0 (44.9–81.7)	58.8 (40.7–77.7)	3.2 (2.0–5.1)			
≥4.70	2020 (33.5)	66.0 (50.5–84.1)	62.3 (47.2–80.1)	3.5 (2.3–5.2)			
Calcium (mg)					<0.001	<0.001	<0.001
<617.0	2005 (33.3)	58.5 (42.0–78.2)	53.3 (36.9–72.8)	3.0 (1.8–4.7)			
617.0–975.84	2014 (33.4)	63.7 (47.0–83.3)	59.8 (42.6–78.8)	3.4 (2.1–5.0)			
≥975.84	2007 (33.3)	64.2 (48.6–81.4)	61.3 (45.4–78.7)	3.5 (2.3–5.3)			

Abbreviation: IQR, interquartile range. ^a^ Percentage values are weighted.

**Table 2:** Odds Ratios of HOMA-IR by 25-hydroxyvitamin D Concentrations.

		With Insulin Resistance/Without Insulin Resistance	70th vs. 30th Percentile	80th vs. 20th Percentile
25-hydroxyvitamin D (nmol/L)		60.5/65.8	47.5/80.7	40.3/88.8
Model 1 ^a^	1 [Reference]	0.32 (0.24–0.43)	0.34 (0.25–0.46)	0.36 (0.26–0.49)
Model 2 ^b^	1 [Reference]	0.56 (0.40–0.78)	0.58 (0.41–0.82)	0.59 (0.41–0.86)
Model 3 ^c^	1 [Reference]	0.57 (0.41–0.80)	0.58 (0.40–0.83)	0.61 (0.42–0.90)
Model 4 ^d^	1 [Reference]	0.61 (0.43–0.86)	0.62 (0.43–0.90)	0.67 (0.45–0.99)
25-hydroxyvitamin D3 (nmol/L)		56.1/62.3	43.4/77.2	35.8/85.4
Model 1 ^a^	1 [Reference]	0.34 (0.26–0.44)	0.35 (0.27–0.46)	0.35 (0.27–0.47)
Model 2 ^b^	1 [Reference]	0.63 (0.46–0.85)	0.65 (0.47–0.90)	0.66 (0.47–0.93)
Model 3 ^c^	1 [Reference]	0.64 (0.47–0.86)	0.66 (0.48–0.92)	0.70 (0.49–0.98)
Model 4 ^d^	1 [Reference]	0.67 (0.49–0.91)	0.71 (0.51–0.99)	0.75 (0.53–1.08)
3-epi-25-hydroxyvitamin D3 (nmol/L)		3.2/3.9	2.2/4.7	1.7/5.9
Model 1 ^a^	1 [Reference]	0.64 (0.53–0.77)	0.51 (0.41–0.63)	0.60 (0.49–0.73)
Model 2 ^b^	1 [Reference]	0.99 (0.80–1.24)	0.81 (0.63–1.04)	0.92 (0.72–1.18)
Model 3 ^c^	1 [Reference]	1.00 (0.82–1.26)	0.82 (0.64–1.05)	0.96 (0.75–1.23)
Model 4 ^d^	1 [Reference]	1.05 (0.92–1.18)	0.85 (0.66–1.10)	1.00 (0.78–1.28)

^a^ Model 1 is shown as the odds ratio (95% confidence interval); adjusted for sex, age, race and ethnicity, and education status. ^b^ Model 2 is shown as an odds ratio (95% confidence interval); further adjusted for body mass index (calculated as weight in kilograms divided by height in meters squared), diabetes, and drinking and smoking. ^c^ Model 3 is shown as odds ratio (95% confidence interval); further adjusted for creatinine(urine), albumin(urine), and cholesterol. ^d^ Model 4 is shown as odds ratio (95% confidence interval); further adjusted for carbohydrate, protein, fat, dietary fiber, vitamin D, calcium.

**Table 3:** Odds Ratio of Insulin resistance by 25-hydroxyvitamin D Concentrations.

	Tertile 1	Tertile 2	Tertile 3	*p* Value for Trend ^a^
Total 25-hydroxyvitamin D (nmol/L)	<51.5	51.5 to 74.7	>74.7	
With Insulin resistance/without Insulin resistance, No.	1324/669	1238/807	1102/886	
Model 1 ^b^	1 [Reference]	0.76 (0.67–0.87)	0.59 (0.52–0.68)	<0.001
Model 2 ^c^	1 [Reference]	0.82 (0.71–0.95)	0.76 (0.65–0.89)	<0.001
Model 3 ^d^	1 [Reference]	0.82 (0.71–0.96)	0.77 (0.66–0.90)	<0.001
Model 4 ^e^	1 [Reference]	0.84 (0.72–0.97)	0.79 (0.67–0.93)	<0.001
25-hydroxyvitamin D3 (nmol/L)	<47.4	47.4 to 70.0	>70.0	
With Insulin resistance/without Insulin resistance, No.	1324/665	1391/918	949/779	
Model 1 ^b^	1 [Reference]	0.77 (0.67–0.88)	0.58 (0.51–0.67)	<0.001
Model 2 ^c^	1 [Reference]	0.84 (0.73–0.98)	0.77 (0.66–0.90)	<0.001
Model 3 ^d^	1 [Reference]	0.85 (0.73–0.99)	0.78 (0.66–0.91)	<0.001
Model 4 ^e^	1 [Reference]	0.86 (0.74–1.00)	0.79 (0.68–0.93)	<0.001
3-epi-25-hydroxyvitamin D3 (nmol/L)	<2.2	2.2 to 4.0	>4.0	
With Insulin resistance/without Insulin resistance, No.	1093/613	1253/821	1318/928	
Model 1 ^b^	1 [Reference]	0.83 (0.73–0.95)	0.77 (0.68–0.88)	<0.001
Model 2 ^c^	1 [Reference]	0.96 (0.83–1.11)	1.05 (0.90–1.20)	<0.001
Model 3 ^d^	1 [Reference]	0.97 (0.83–1.12)	1.06 (0.91–1.23)	<0.001
Model 4 ^e^	1 [Reference]	0.98 (0.84–1.14)	1.08 (0.92–1.26)	<0.001

^a^ *p* value for trend based on log-transformed total 25-hydroxyvitamin D, 25-hydroxyvitamin D3, and 3-epi-25-hydroxyvitamin D3 concentrations. ^b^ Model 1 is shown as odds ratio (95% confidence interval); adjusted for sex, age, race and ethnicity, and education status. ^c^ Model 2 is shown as odds ratio (95% confidence interval); further adjusted for body mass index (calculated as weight in kilograms divided by height in meters squared), diabetes, and drinking and smoking. ^d^ Model 3 is shown as odds ratio (95% confidence interval); further adjusted for creatinine (urine), albumin(urine), and cholesterol. ^e^ Model 4 is shown as odds ratio (95% confidence interval); further adjusted for carbohydrate, protein, fat, dietary fiber, vitamin D, calcium.

## Data Availability

Some or all data, models, or code generated or used during the study are available from the corresponding author.

## References

[1] Binkley N., Ramamurthy R., Krueger D. (2012). Low vitamin D status: Definition, prevalence, consequences, and correction. Rheum. Dis. Clin. N. Am..

[2] Krist A.H., Davidson K.W., Mangione C.M., Cabana M., Caughey A.B., Davis E.M., Donahue K.E., Doubeni C.A., Epling J.W., Kubik M. (2021). Screening for Vitamin D Deficiency in Adults: US Preventive Services Task Force Recommendation Statement. JAMA.

[3] Mithal A., Wahl D.A., Bonjour J.P., Dawson-Hughes B., Eisman J.A., Fuleihan G.E., Josse R.G., Lips P., Morales-Torres J., IOF Committee of Scientific Advisors (CSA) Nutrition Working Group (2009). Global vitamin D status and determinants of hypovitaminosis D. Osteoporos. Int..

[4] Souberbielle J.-C., Body J.-J., Lappe J.M., Plebani M., Shoenfeld Y., Wang T.J., Bischoff-Ferrari H.A., Cavalier E., Ebeling P.R., Fardellone P. (2010). Vitamin D and musculoskeletal health, cardiovascular disease, autoimmunity and cancer: Recommendations for clinical practice. Autoimmun. Rev..

[5] Muscogiuri G., Altieri B., Annweiler C., Balercia G., Pal H.B., Boucher B.J., Cannell J.J., Foresta C., Grübler M.R., Kotsa K. (2017). Vitamin D and chronic diseases: The current state of the art. Arch. Toxicol..

[6] Barragan M., Good M., Kolls J.K. (2015). Regulation of Dendritic Cell Function by Vitamin D. Nutrients.

[7] Pittas A.G., Jorde R., Kawahara T., Dawson-Hughes B. (2020). Vitamin D Supplementation for Prevention of Type 2 Diabetes Mellitus: To D or Not to D?. J. Clin. Endocrinol. Metab..

[8] Sacerdote A., Dave P., Lokshin V., Bahtiyar G. (2019). Type 2 Diabetes Mellitus, Insulin Resistance, and Vitamin D. Curr. Diab. Rep..

[9] Gulseth H.L., Wium C., Angel K., Eriksen E.F., Birkeland K.I. (2017). Effects of Vitamin D Supplementation on Insulin Sensitivity and Insulin Secretion in Subjects with Type 2 Diabetes and Vitamin D Deficiency: A Randomized Controlled Trial. Diabetes Care.

[10] Wang X., Zhai M., Ren Z., Ren H., Li M., Quan D., Chen L., Qiu L. (2021). Exploratory study on classification of diabetes mellitus through a combined Random Forest Classifier. BMC Med. Inform. Decis. Mak..

[11] Whiting D.R., Guariguata L., Weil C., Shaw J. (2011). IDF diabetes atlas: Global estimates of the prevalence of diabetes for 2011 and 2030. Diabetes Res. Clin. Pract..

[12] Guariguata L., Whiting D., Weil C., Unwin N. (2011). The International Diabetes Federation diabetes atlas methodology for estimating global and national prevalence of diabetes in adults. Diabetes Res. Clin. Pract..

[13] Onyango A.N. (2018). Cellular Stresses and Stress Responses in the Pathogenesis of Insulin Resistance. Oxid Med. Cell. Longev..

[14] Simonson G.D., Kendall D.M. (2005). Diagnosis of insulin resistance and associated syndromes: The spectrum from the metabolic syndrome to type 2 diabetes mellitus. Coron. Artery. Dis..

[15] Barchetta I., De Bernardinis M., Capoccia D., Baroni M.G., Fontana M., Fraioli A., Morini S., Leonetti F., Cavallo M.G. (2013). Hypovitaminosis D is independently associated with metabolic syndrome in obese patients. PLoS ONE.

[16] Olson M.L., Maalouf N.M., Oden J.D., White P.C., Hutchison M.R. (2012). Vitamin D deficiency in obese children and its relationship to glucose homeostasis. J. Clin. Endocrinol. Metab..

[17] Kayaniyil S., Retnakaran R., Harris S.B., Vieth R., Knight J.A., Gerstein H.C., Perkins B.A., Zinman B., Hanley A.J. (2011). Prospective associations of vitamin D with beta-cell function and glycemia: The PROspective Metabolism and ISlet cell Evaluation (PROMISE) cohort study. Diabetes.

[18] Dunlop T.W., Vaisanen S., Frank C., Molnár F., Sinkkonen L., Carlberg C. (2005). The human peroxisome proliferator-activated receptor delta gene is a primary target of 1alpha,25-dihydroxyvitamin D3 and its nuclear receptor. J. Mol. Biol..

[19] Nazarian S., St P.J., Boston R.C., Jones S.A., Mariash C.N. (2011). Vitamin D3 supplementation improves insulin sensitivity in subjects with impaired fasting glucose. Transl. Res..

[20] Bourlon P.M., Billaudel B., Faure-Dussert A. (1999). Influence of vitamin D3 deficiency and 1,25 dihydroxyvitamin D3 on de novo insulin biosynthesis in the islets of the rat endocrine pancreas. J. Endocrinol..

[21] Forno E., Han Y.Y., Muzumdar R.H., Celedón J.C. (2015). Insulin resistance, metabolic syndrome, and lung function in US adolescents with and without asthma. J. Allergy. Clin. Immunol..

[22] Agarwal S., Fulgoni V.R. (2021). Intake of Potatoes Is Associated with Higher Diet Quality, and Improved Nutrient Intake and Adequacy among US Adolescents: NHANES 2001-2018 Analysis. Nutrients.

[23] Peng Q., Harlow S.D., Park S.K. (2015). Urinary arsenic and insulin resistance in US adolescents. Int. J. Hyg. Environ. Health.

[24] Matli B., Schulz A., Koeck T., Falter T., Lotz J., Rossmann H., Pfeiffer N., Beutel M., Münzel T., Strauch K. (2021). Distribution of HOMA-IR in a population-based cohort and proposal for reference intervals. Clin. Chem. Lab. Med..

[25] Shashaj B., Luciano R., Contoli B., Morino G.S., Spreghini M.R., Rustico C., Sforza R.W., Dallapiccola B., Manco M. (2016). Reference ranges of HOMA-IR in normal-weight and obese young Caucasians. Acta Diabetol..

[26] Wang Z., Wang Y.J., Liu Z.Y., Li Q., Kong Y.W., Chen Y.W., Sun Y.H., Dong J.Z. (2022). Effect of Insulin Resistance on Recurrence after Radiofrequency Catheter Ablation in Patients with Atrial Fibrillation. Cardiovasc. Drugs Ther..

[27] Qi T., Xueqin L., Peipei S., Lingzhong X. (2015). Optimal cut-off values for the homeostasis model assessment of insulin resistance (HOMA-IR) and pre-diabetes screening: Developments in research and prospects for the future. Drug Discov. Ther..

[28] Li D., Wei H., Xue H., Zhang J., Chen M., Gong Y., Cheng G. (2018). Higher serum 25(OH)D level is associated with decreased risk of impairment of glucose homeostasis: Data from Southwest China. BMC Endocr. Disord..

[29] Gao Y., Zheng T., Ran X., Ren Y., Chen T., Zhong L., Yan D., Yan F., Wu Q., Tia H. (2018). Vitamin D and Incidence of Prediabetes or Type 2 Diabetes: A Four-Year Follow-Up Community-Based Study. Dis. Markers.

[30] Niroomand M., Fotouhi A., Irannejad N., Hosseinpanah F. (2018). Does high-dose vitamin D supplementation impact insulin resistance and risk of development of diabetes in patients with pre-diabetes? A double-blind randomized clinical trial. Diabetes Res. Clin. Pract..

[31] Pilz S., van den Hurk K., Nijpels G., Stehouwer C.D., Van’t Riet E., Kienreich K., Tomaschitz A., Dekker J.M. (2012). Vitamin D status, incident diabetes and prospective changes in glucose metabolism in older subjects: The Hoorn study. Nutr. Metab. Cardiovasc. Dis..

[32] Heshmat R., Tabatabaei-Malazy O., Abbaszadeh-Ahranjani S., Shahbazi S., Khooshehchin G., Bandarian F., Larijani B. (2012). Effect of vitamin D on insulin resistance and anthropometric parameters in Type 2 diabetes; a randomized double-blind clinical trial. Daru J. Fac. Pharm. Tehran Univ. Med. Sci..

[33] Lin C.A., Liu Y.P., Chen Y.C., Yu W., Xiong X.J., Huang H.Y., Li W.C., Chen J.Y. (2021). Gender-specific and age-specific associations of the homoeostasis model assessment for IR (HOMA-IR) with albuminuria and renal function impairment: A retrospective cross-sectional study in Southeast China. BMJ Open.

[34] Isokuortti E., Zhou Y., Peltonen M., Bugianesi E., Clement K., Bonnefont-Rousselot D., Lacorte J.M., Gastaldelli A., Schuppan D., Schattenberg J.M. (2017). Use of HOMA-IR to diagnose non-alcoholic fatty liver disease: A population-based and inter-laboratory study. Diabetologia.

[35] Lee C.H., Shih A.Z., Woo Y.C., Fong C.H., Leung O.Y., Janus E., Cheung B.M., Lam K.S. (2016). Optimal Cut-Offs of Homeostasis Model Assessment of Insulin Resistance (HOMA-IR) to Identify Dysglycemia and Type 2 Diabetes Mellitus: A 15-Year Prospective Study in Chinese. PLoS ONE.

[36] Paolisso G., Tagliamonte M.R., Rizzo M.R., Giugliano D. (1999). Advancing age and insulin resistance: New facts about an ancient history. Eur. J. Clin. Investig..

[37] Gayoso-Diz P., Otero-González A., Rodriguez-Alvarez M.X., Gude F., García F., De Francisco A., Quintela A.G. (2013). Insulin resistance (HOMA-IR) cut-off values and the metabolic syndrome in a general adult population: Effect of gender and age: EPIRCE cross-sectional study. BMC Endocr. Disord..

[38] Gluvic Z., Zaric B., Resanovic I., Obradovic M., Mitrovic A., Radak D., Isenovic E.R. (2017). Link between Metabolic Syndrome and Insulin Resistance. Curr. Vasc. Pharm..

[39] Yang Y., Hu X., Zhang Q., Zou R. (2016). Diabetes mellitus and risk of falls in older adults: A systematic review and meta-analysis. Age Ageing.

[40] Tseng M., Fang C.Y. (2015). Acculturation and Insulin Resistance among US Chinese Immigrant Women. Ethn. Dis..

[41] Rosenberg D.E., Jabbour S.A., Goldstein B.J. (2005). Insulin resistance, diabetes and cardiovascular risk: Approaches to treatment. Diabetes Obes. Metab..

[42] Papakonstantinou E., Oikonomou C., Nychas G., Dimitriadis G.D. (2022). Effects of Diet, Lifestyle, Chrononutrition and Alternative Dietary Interventions on Postprandial Glycemia and Insulin Resistance. Nutrients.

[43] Kahleova H., Hlozkova A., Fleeman R., Fletcher K., Holubkov R., Barnard N.D. (2019). Fat Quantity and Quality, as Part of a Low-Fat, Vegan Diet, Are Associated with Changes in Body Composition, Insulin Resistance, and Insulin Secretion. A 16-Week Randomized Controlled Trial. Nutrients.

[44] Boucher B.J., John W.G., Noonan K. (2004). Hypovitaminosis D is associated with insulin resistance and beta cell dysfunction. Am. J. Clin. Nutr..

[45] Kumar S., Davies M., Zakaria Y., Mawer E.B., Gordon C., Olukoga A.O., Boulton A.J. (1994). Improvement in glucose tolerance and beta-cell function in a patient with vitamin D deficiency during treatment with vitamin D. Postgrad. Med. J..

[46] Pramono A., Jocken J.W.E., Blaak E.E., van Baak M.A. (2020). The Effect of Vitamin D Supplementation on Insulin Sensitivity: A Systematic Review and Meta-analysis. Diabetes Care.

[47] Gallagher J.C., Yalamanchili V., Smith L.M. (2013). The effect of vitamin D supplementation on serum 25OHD in thin and obese women. J. Steroid Biochem. Mol. Biol..

[48] Pereira-Santos M., Costa P.R., Assis A.M., Santos C.A., Santos D.B. (2015). Obesity and vitamin D deficiency: A systematic review and meta-analysis. Obes. Rev. Off. J. Int. Assoc. Study Obes..

[49] Wright C.S., Weinheimer-Haus E.M., Fleet J.C., Peacock M., Campbell W.W. (2015). The Apparent Relation between Plasma 25-Hydroxyvitamin D and Insulin Resistance is Largely Attributable to Central Adiposity in Overweight and Obese Adults. J. Nutr..

[50] Nsiah-Kumi P.A., Erickson J.M., Beals J.L., Ogle E.A., Whiting M., Brushbreaker C., Borgeson C.D., Qiu F., Yu F., Larsen J.L. (2012). Vitamin D Insufficiency Is Associated with Diabetes Risk in Native American Children. Clin. Pediatr..

[51] Tao S., Yuan Q., Mao L., Chen F.L., Ji F., Cui Z.H. (2017). Vitamin D deficiency causes insulin resistance by provoking oxidative stress in hepatocytes. Oncotarget.

[52] Rasic-Milutinovic Z., Perunicic-Pekovic G., Pljesa S. (2000). Clinical significance and pathogenic mechanisms of insulin resistance in chronic renal insufficiency (part II): Pathogenic factors of insulin resistance in chronic renal insufficiency. Med. Pregl..

[53] Dorothy T., Shawn S.D. (2009). Vitamin D: Emerging new roles in insulin sensitivity. Nutr. Res. Rev..

[54] Tai K., Need A.G., Horowitz M., Chapman I.M. (2007). Vitamin D, glucose, insulin, and insulin sensitivity. Nutrition.

[55] Santos L.R.d., Lima A.G.A., Braz A.F., Melo S.R.D., Morais J.B.S., Severo J.S., de Oliveira A.R.S., Cruz K.J.C., Marreiro D.D.N. (2017). Role of vitamin D in insulin resistance in obese individuals. Nutrire.

[56] Sung C.C., Liao M.T., Lu K.C., Wu C.C. (2012). Role of Vitamin D in Insulin Resistance. J. Biomed. Biotechnol..

